# Bleeding Disorders and Dental Implants: Review and Clinical Indications

**DOI:** 10.3390/jcm12144757

**Published:** 2023-07-18

**Authors:** Christian Bacci, Claudia Schiazzano, Ezio Zanon, Edoardo Stellini, Luca Sbricoli

**Affiliations:** 1 Department of Neurosciences, School of Dentistry, University of Padua, 35128 Padua, Italy; claudiaschiazzano95@gmail.com (C.S.); edoardo.stellini@unipd.it (E.S.); luca.sbricoli@unipd.it (L.S.); 2 Haemophilia Centre, General Medicine, Padua University Hospital, 35128 Padua, Italy; ezio.zanon@unipd.it

**Keywords:** dental implants, bleeding disorders, haemophilia, von Willebrand disease

## Abstract

Background: Bleeding disorders can be divided into three categories: congenital coagulation disorders (CCDs), antiplatelet-induced bleeding disorders (APBDs) and anticoagulant-induced bleeding disorders (ACBDs). Implant placement can be challenging in these kinds of patients. The aim of this study is to provide evidence on implant surgery in patients with bleeding disorders and to generate some practical recommendations for clinicians. Material and Methods: Pubmed/MEDLINE, Scopus, Web of Science and Cochrane Library databases were screened. The latest search was performed in July 2022. Case reports, case series, cohort studies, cross-sectional studies, case control studies, reviews, consensus reports, surveys and animal studies were included in the analysis. Results: Seventeen articles on CCDs were found, fourteen on APBDs and twenty-six on ACBDs. Most of these articles were case reports or case series. Patients with CCDs can be treated after the infusion of the missing coagulation factor. Patients with APBDs can be treated without withdrawing the therapy. Patients with ACBDs should be treated depending on the anticoagulative medication. Conclusion: Despite the low level of evidence, dental implants can be safely placed in patients with bleeding disorders. However, careful preoperative evaluation and the adoption of local and post-operative bleeding control measures are mandatory.

## 1. Introduction

Bleeding disorders are a group of conditions when blood cannot clot properly. They can be congenital or acquired. The congenital ones are inherited and are quite rare. The acquired ones can develop from a pathological condition or can be caused by the intake of drugs belonging to the category of antiplatelet or to the inhibitors of coagulation factors.

Among congenital bleeding disorders, the most common is von Willebrand disease, caused by diminished quantity or by a structural defect of the von Willebrand factor [1]. Von Willebrand disease is primarily a hereditary disease, but an acquired variant of von Willebrand disease has also been observed. The most famous classification of the disease distinguishes three inherited types: classes 1 and 3 include quantitative factor deficits, while class 2 includes qualitative defects [1,2].

The second most common congenital bleeding disorder is haemophilia, a class of hereditary diseases whose etiopathogenetic mechanism is based on defects of the proteins involved in the coagulation process. It was once called “the royal disease” due to its presence in European royal families in the 19th and 20th centuries. Queen Victoria of England (1818–1901) was an asymptomatic carrier and her descendants in turn transmitted the mutation in various royal houses across Europe [3,4,5].

Antiplatelet drugs, also called “antiaggregant”, interfere with the platelet plug formation. Acetylsalicylic acid (aspirin) and clopidogrel are the most commonly prescribed. They are used both in acute treatment and in the prevention of coronary artery disorders and stroke; they are also used for prevention of venous thromboembolism after orthopedic surgery, in generic vascular diseases, unstable angina and in patients who have undergone percutaneous coronary artery surgery and cardiac surgery [6].

Coagulation inhibitors selectively act on certain clotting factors, hindering the clot formation process. These drugs are indicated for deep venous thrombosis, venous and arterial thromboembolic disease, pulmonary embolism, atrial fibrillation with risk of embolization, mechanical heart valve prostheses (to prevent thrombus formation on valves), myocardial infarction, recent heart attack to prevent the onset of new cardiovascular events (another heart attack, stroke, etc.), unstable angina, acute peripheral arterial occlusion and unstable coronary syndromes [7]. Anticoagulant medicines that have been most used over the years are vitamin K-dependent inhibitor drugs Warfarin [8] and Acenocumarol. Recently, to overcome the limits of these kind of anticoagulants, direct thrombin inhibitors and activated Factor X inhibitors have been proposed [9].

Another anticoagulant worth mentioning is heparin, although is it most often used as short-term therapy or to overlap a long-term therapy when a suspension is required [10]. Thrombocytopenia goes beyond the boundaries of the above classification, as it can be due to a congenital disorder or medically induced. By definition, thrombocytopenia is observed when the platelet count is below 150,000/μL. The reasons why a platelet count may be below the normal range can be many and varied [4].

Dental implants are a well-established solution for the rehabilitation of edentulous areas, and the number of implants placed worldwide is continuously increasing. Implants have also proven to be an effective solution in patients with systemic diseases [11]. Today, one of the categories of patients where clinicians are reluctant to place implants is patients with bleeding disorders. Hence, they prefer to send the patient to hospital facilities or choose other prosthetic solutions. Moreover, randomized controlled clinical trials cannot be conducted for security and ethical reasons. Therefore, the management of these patients is often determined by expert opinions and practical guidelines that differ between countries. The aim of this paper is to perform a literature review about dental implants inserted in patients with bleeding disorders and to provide indications for clinicians to help them deal with these patients.

## 2. Materials and Methods

### 2.1. Search Strategy

Bibliographic electronic searches were performed in the Pubmed/MEDLINE, Scopus, Web of Science and Cochrane Library databases. The latest research was performed in July 2022. Only articles in English were considered. The search strings used for CCDs, APBDs and ACBDs are reported in the Appendix A section.

The keywords “oral implant”, “dental implant(s)”, “implantology” and “implant(s)” were searched in combination with the following terms: “haemophilia”, “von Willebrand disease”, “thrombocytopenia”, “bleeding disorder(s)”, “aspirin”, “clopidogrel”, “prasugrel”, “ticagrelor”, “anti-platelet”, “antiaggregant”, “warfarin”, “sintrom”, “heparin”, “dabigatran”, “rivaroxaban”, “apixaban”, “edoxaban”, “anticoagulant”, “NOAC”, “DOAC”, “novel anticoagulant”, and “direct anticoagulant”.

### 2.2. Inclusion and Exclusion Criteria

Randomized controlled trials, cohort studies, cross-sectional studies, case-control studies, review, case series, case reports and animal studies were included in the analysis. Letters to the editor, expert opinion and article evaluations were excluded. Only articles in English and with the full text available were considered.

## 3. Results

**Figure 1:**
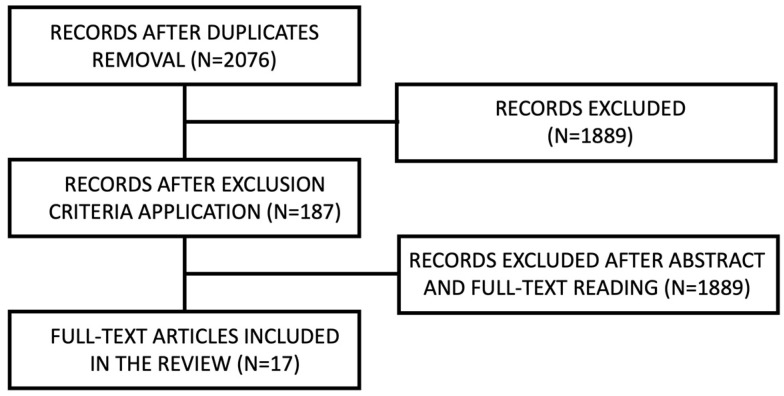
CCD search diagram.

**Figure 2:**
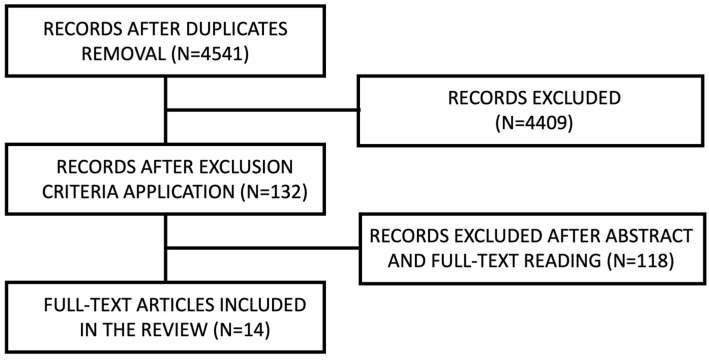
APBD search diagram.

**Figure 3:**
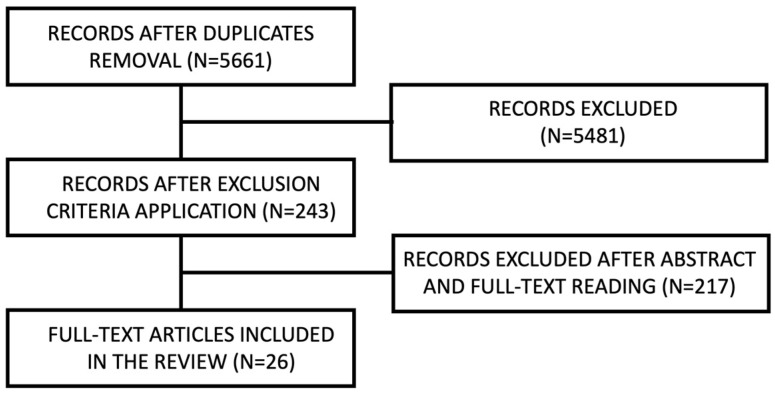
ACBD search diagram.

A flow diagram describing the web search strategy is presented below. Search results are reported as a diagram in Figure 1, Figure 2 and Figure 3 for CCDs, APBDs and ACBDs, respectively.

Among the articles found on PubMed and Scopus, seventeen were selected for the first section, fourteen for the second and twenty-six for the third. Six articles considered patients with either anticoagulant or antiplatelet therapy. Therefore, they were considered for analysis in both the second and the third categories. Articles retrieved from the electronic search are reported in Table A1, Table A2 and Table A3. Literature reviews were not reported. Results are divided into three sections, one for each of the different bleeding disorders.

### 3.1. Congenital Coagulation Disorders (CCDs)

Among seventeen articles, twelve were case reports and five reviews. Seven articles considered patients with haemophilia [12,13,14,15,16,17,18], seven considered patients with von Willebrand disease [19,20,21,22], one considered patients with idiopathic thrombocytopenic purpura [23]. Five articles performed the surgery flapless [12,18,19,20,21], three articles did not [13,14,17] and four articles did not specify [15,16,22,23]. One article did not report on post-operative complications [22], one article reported mild post-operative bleeding complications [18] and one article reported a severe bleeding complication, which required an emergency tracheotomy and hospitalization of the patient [23]. The remaining articles reported no complications [12,13,14,15,16,17,19,20,21].

### 3.2. Antiplatelet-Induced Bleeding Disorders (APBDs)

The fourteen articles included one case report [24], one case-cross-over study [25], two cohort studies [26,27], four case-control studies [28,29,30,31], two animal studies [32,33] and four reviews [34,35,36,37]. In almost all clinical studies related to this section, implant surgeries took place without interruption of the antiaggregative therapy [24,26,27,28,29,30,31]. Only two studies reported a change in drug therapy [25,26]. This occurred in patients with dual antiaggregant therapy or when an anticoagulant was used in addition. Few hemorrhagic complications were observed, with a range between 1% and 15% of the surgeries. Most of the studies agreed on compressive hemostasis after surgery, with or without the association of tranexamic acid.

### 3.3. Anticoagulant-Induced Bleeding Disorders (ACBD)

Among twenty-six articles, four were case reports [38,39,40,41], one was a case series [42], six were cohort studies [8,26,43,44,45,46], eight were case control studies [28,29,30,31,47,48,49,50], six were reviews [35,51,52,53,54,55] and one was an animal study. Considering the clinical studies, five authors treated patients undergoing conventional anticoagulant drugs [8,39,40,43,48], six treated patients using DOAC [38,42,44,46,49,50] and, in the remaining articles, the sample considered used both medications [26,28,29,30,31,47]. Case–control studies showed no significant differences in hemorrhagic complications after implant surgery between patients in the test and in the control group. However, in every clinical study, where a hemorrhagic complication occurred, it was always solved with only the help of local hemostatic measurements [28,29,31,38,43,44,46,47,48,49,50]. The only animal study investigated the effects of rivaroxaban on osteointegration, and it showed that it did not affect it.

## 4. Discussion

The present review addressed implant rehabilitation of patients with bleeding disorders. The information obtained from the analysis of the clinical studies was rather heterogeneous, and thus difficult to compare with each other.

As regards Section 1, in almost all the case reports, local hemostatic measures and pre-medication with missing factors or desmopressin were implemented. It did not happen in the case report concerning the patient with idiopathic thrombocytopenic purpura [23]. It may not be a coincidence that this case report is the only one in which the patient developed complications not manageable by solely local hemostatic measures or the simple administration of tranexamic acid.

Literature reviews considered the safety of oral surgery in general and not specifically implant surgery. Four out of five reviews concluded that there is no contraindication to implant surgery in this type of patient [56,57,58]. The only article that advised against implant therapy in patients with congenital coagulation defects was that of Hwang et al. [59]. According to the authors, in fact, elective procedures such as implant rehabilitation should not be performed unless hemostasis can be assured with certainty at the end of the procedure. The statement is reinforced by pointing out that there are alternatives to implant rehabilitation that do not involve a surgical phase such as, for example, removable prostheses. A common consideration in all reviews is to take extreme care not to perforate the lingual cortical during surgery, as bleeding of the vasal plexus in that area would result in major bleeding that would be difficult to resolve.

In Section 2, evidence emerged of the non-discontinuation of antiplatelet therapy, even if dual. This attitude, according to the review by Cervino et al., is of recent acquisition by clinicians [36]. Previously, there was a tendency to have antiplatelet therapy arbitrarily suspended for a few days before therapy. Moreover, the same review points out that suspension for a few days is not at all effective in decreasing bleeding risk and, indeed, subjects the patient to unnecessary thromboembolic risk. All the literature, therefore, agrees that antiplatelet therapy should not be discontinued [34,35,36,37]. The only major bleeding reported in the literature following dental implant placement in a patient who had not discontinued antiplatelet therapy was resolved with local hemostatic measures, without requiring further intervention [34].

In Section 3, articles concerning implant placement in patients taking anticoagulant drugs were considered. In this category, it is important to distinguish between conventional drugs and DOACs. It turned out, in fact, that the former should never be discontinued, but an evaluation of INR values should be carried out, making sure that they are compatible with surgery. In the second case, however, different attitudes emerged from clinicians. Some authors considered the discontinuation of direct anticoagulants as unnecessary, while others recommended their discontinuation at least 24 h in advance. This uncertainty is surely due to the absence of international guidelines on this topic. Previous reviews did not help to settle doubts because they refer to surgery in general on patients taking anticoagulant drugs [51,52,53,54,55]. Specifically, the Madrid review by Madrid and Sanz presented evidence on only one case of implantology, compared with numerous cases of dental extractions [51]. The review by Sivolella et al. also focused more on dental extractions, but important indications were reported about the use of local hemostatic measures, which are in agreement with the present review.

In light of the findings of this narrative review, it is possible to state that most indications for implant surgery are derived from extractive surgery, where more of the literature is present.

## 5. Conclusions

The limited evidence about this topic cannot be considered strong, since these recommendations are based on case reports, case series, a few cohort studies and some reviews. The present review may be helpful for clinicians who find themselves treating patients with this type of condition. Accurate medical history taking, proper patient information, close collaboration with hematologists and thorough application of peri-operative hemostasis control protocols can be considered the key factors in avoiding possible serious complications. More clinical trials should be performed on this argument in the future to achieve a higher level of scientific evidence.

### Clinical Importance

The following clinical indications should be considered when treating patients with bleeding disorders.

1. Consulting the physician who treat the patient for their bleeding disorder before implant surgery;
2. Almost every study included in the present review suggests administering deficiency factor or desmopressin before the surgery in patients with congenital hemostasis disease;
3. Do not withdraw antiaggregative therapy for implant surgery, even if dual; only one study suspended the antiaggregative therapy, but it was due to a concomitant anticoagulant therapy. There is no evidence for the beneficial effect of suspending antiaggregative therapy.
4. Do not interrupt therapy with dicumarolics. On the contrary, clinicians should check the INR value before implant surgery and treat any bleeding complications with local hemostatic measures.
5. When dealing with direct anticoagulants (DAOC) skip only one dose of the drug, the day before;
6. Adopt local hemostatic measures at the end of the procedure and in case of postoperative bleeding: compressive hemostasis with gauze soaked in tranexamic acid showed excellent results in both situations. Regardless of the type of bleeding disorder, all authors agreed in suggesting the application of local hemostatic measures at the end of surgery;
7. Instruct the patient about the appropriate post-operative measures: soft and cold diet for 2–3 days, avoiding vigorous rinses, physical effort and the supine position, applying gauze for 5 min in case of bleeding. If these are not enough, repeat the application of the gauze soaked with tranexamic acid. Not all articles reported postoperative instructions, but those that did agree with what is reported here.

## Data Availability

Not applicable.

## Appendix A

**Table A1:** Published evidence about dental implants performed in patients with CCDs.

	Study Type	Underlying Blood Disease	No. of Implants Inserted for Intervention	Flapless	Prophylaxis	Additional Drug Administered after the Procedure	Local Haemostatic Measurements	Bleeding Complications
Gornitisky et al., 2005 [16]	Case report	Moderate haemophilia A	3; 2; 1	Unspecified	Factor VIII; Tranexamic acid per os	Factor VIII; Tranexamic acid per os	Suture	No
Rosen et al., 2005 [15]	Case report	Moderate haemophilia A	3; 2; 1	Unspecified	Unspecified	Unspecified	Unspecified	No
Neskoromna-Jȩdrzejczak et al., 2018 [17]	Case report	Severe haemophilia B	2; 2	No	Recombinant Factor IX	Recombinant Factor IX	Suture	No
Castellanos-Cosano et al., 2014 [13]	Case report	Severe haemophilia A; HIV; HCV	3; 2	No	Antibiotic; Factor VIII (FVIII), Tranexamic acid per os	Antibiotic; Factor VIII (FVIII), Tranexamic acid per os	Unspecified	No
Fénelon et al., 2017 [20]	Case report	Von Willebrand disease	1	Yes	Von Willebrand Factor (vWF)	Von Willebrand Factor (vWF); Antibiotic	Suture; fibrin glue	No
Kang et al., 2018 [19]	Case report	Von Willebrand disease	1	Yes	Desmopressin	none	Unspecified	No
Sung-Tak Lee et al., 2018 [23]	Case report	Porpora trombocitopenica idiopatica	1	Presumably not	none	none	Unspecified	Severe
Calvo-Guirado et al., 2019 [14]	Case report	Severe haemophilia B	1	No	Antibiotic; Factor IX; Tranexamic acid per os	Factor IX; Tranexamic acid per os and rinses	Suture; particulate bone graft and collagen membrane soaked in tranexamic acid; compressive hemostasis with gauze soaked in tranexamic acid	No
Bacci et al., 2021 [21]	Case report	Von Willebrand disease type 2B	5	Yes	Factor VIII + VWF; tranexamic acid	tranexamic acid per os;	Unspecified	No
Bacci et al., 2021 [12]	Case report	Mild haemophilia A	1	Yes	Factor VIII; tranexamic acid	Unspecified	Unspecified	No
Takashima et al., 2021 [22]	Case report	Von Willebrand disease type 1	6	Unspecified	Factor VIII + VWF	Unspecified	Unspecified	Unspecified
Kinalsky et al., 2021 [18]	Case report	Haemophilia A	3; 3	Yes	Factor VIII; tranexamic acid	no	Suture	Mild

**Table A2:** Published evidence about dental implants performed in patients with APBDs.

	Study Type	Antithrombotic Treatment	Discontinuation of the Pharmacological Therapy	No. of Procedures	Post-Operative Bleeding Complications	Management of Complications
Flanagan et al., 2015 [24]	Case report	Clopidogrel + ASA	No	1	1	Compressive hemostasis with sponge soaked in tranexamic acid
Clemm et al., 2015 [28]	Case–control study	Dicumarols (32)/bridging with LMWH (8)/Dabigatran (6)/Rivaroxaban (8)/Apixaban (2)/Antiaggregants (61)	No	61 (antiaggregants)	4 (1 antiaggregant; 2 dicumarols e 1 dicumarols bridged with LMWH)	Compressive hemostasis with gauze soaked in tranexamic acid/Compressive hemostasis with gauze soaked in tranexamic acid + additional suture/revision of the wound and electrocoagulation
Tabrizi et al., 2018 [25]	Case-crossover study	Clopidogrel/ASA	Only in the second session of the study	41	/	/
Rubino et al., 2019 [26]	Retrospective cohort study	ASA/Clopidogrel/Warfarin/DOAC/ASA + clopidogrel /Clopidogrel + warfarin/ASA + clopidogrel + warfarin/ASA + clopidogrel + DOAC/ASA + DOAC/ASA + warfarin + DOAC	Only in 4 cases, after consulting the physician	218	2 (1 in ASA + warfarin; 1 in warfarin)	Cauterization and infiltration with lidocaine
Kaura et al., 2021 [27]	Prospective cohort study	Clopidogrel/ASA/Clopidogrel + ASA	No	65	1 (in dual therapy)	/
Manor et al., 2021 [29]	Case–control study	Clopidogrel/ASA/DOAC/Warfarin/Combinations	No	72 (*+121 control group*)	4 (1 warfarin + DOAC; 2 Clopidogrel + ASA; 1 warfarin + clopidogrel); *+ 7 control group*	Suture/Suture + Compressive hemostasis with sponge gauze in tranexamic acid
Broekema et al., 2021 [30]	Case–control study	Antiaggregants/Dicumarols	No	8 (*+7 control group*)	0	/
Buchbender et al., 2021 [31]	Case–control study	Antiaggregants/Dicumarols/DOACs	No	95 (+100 control group)	15	Compressive hemostasis with gauze soaked in tranexamic acid

**Table A3:** Published evidence about dental implants performed in patients with ACBDs.

	Study Type	Anticoagulant Therapy	No. of Patients in the Study	No. of implants	Discontinuation of the Pharmacological Therapy	Flapless	Local Hemostatic Measures	Bleeding Complications	Management of Complications
Ferrieri et al., 2007 [8]	Cohort study	Warfarin	3	7	No	No	Suture + compressive hemostasis for 30 min with gauzed soaked in saline	No	/
Bacci et al., 2011 [48]	Case–control study	Warfarin	50	159	No	No	Suture + compressive hemostasis with gauze soaked in tranexamic acid for 30–60 min	2	Compressive hemostasis with gauze soaked in tranexamic acid for 1 h
Miranda et al., 2011 [40]	Case report	Warfarin	1	4	Bridged with heparin	No	Unspecified	No	/
Hong et al., 2012 [43]	Cohort study	Warfarin	1	2	No	Unspecified	“Poncho” of gingival former soaked with tramexamic acid	1	Local hemostatic measures and reinforcement of home care instructions
Broekema et al., 2014 [30]	Case–control study	Antiaggregants/Dicumarols	7	Unspecified	No	No	Unspecified	0	/
Clemm et al., 2015 [28]	Case–control study	Dicumarolici (32)/bridging with LMWH (8)/Dabigatran (6)/Rivaroxaban (8)/Apixaban (2)/Antiaggregants (61)	117 (among them, 61 in therapy with antiaggregants)	Unspecified	No	No	Suture; electrocoagulation	4 (1 antiaggreganti; 2 dicumarolici e 1 dicumarolici embricato con LMWH)	Compressive hemostasis with gauze soaked in tranexamic acid/Compressive hemostasis with gauze soaked in tranexamic acid + additional suture/revision of the wound and electrocoagulation
Gomez-Moreno 2016 [49]	Case–control study	Rivaroxaban	18	43	No	No	Suture + compressive hemostasis with gauze soaked in tranexamic acid	1	Compressive hemostasis with gauze soaked in tranexamic acid
Romero-Ruiz et al., 2015 [41]	Case report	Acenocumarolo	1	12	No	Yes	Unspecified	No	/
Gomez-Moreno 2018 [50]	Case–control study	Dabigatran	29	67	Yes	No	Suture + compressive hemostasis with gauze soaked in tranexamic acid for 30–60 min	2	Compressive hemostasis with gauze soaked in tranexamic acid
Kim et al., 2018 [23]	Case report	Rivaroxaban	1	2	Yes	No	Suture + compressive hemostasis for 1 h	Yes (3)	Compressive hemostasis with gauze + applying oxidized regenerated cellulose
Okamoto et al., 2018 [45]	Cohort study	Unspecified	289	Unspecified	No	Unspecified	Unspecified	0	/
Gandhi et al., 2019 [42]	Case report	Rivaroxaban (3); Apixaban (1); Dabigatran (1)	6	18	No	Yes	Unspecified	No	/
Rubino et al., 2019 [26]	Cohort study	Antiaggrant/Warfarin/DOAC/combinations	176	218	Only in 4 cases	Unspecified	Unspecified	2 (1 in ASA + warfarin; 1 in warfarin)	Local hemostatic measures
Kwak et al., 2019 [46]	Cohort study	Dabigatran (3); Rivaroxaban (3); Apixaban (3)	8	Unspecified	Yes: for 24 h in 8 cases; 48 h for 1 case	No	Suture + compressive hemostasis for 1 h	3	Compressive hemostasis
Al Zoman et al., 2013 [39]	Case report	Warfarin	1	1; 1	No	Yes	Compressive hemostasis	No	/
Sannino et al., 2020 [47]	Case–control study	Warfarin (40) e rivaroxaban (40)	80	320	No	No	Bone wax and spongostan in the extraction site + Compressive hemostasis with gauze soaked in tranexamic acid	Gruppo warfarin: 29 mild, 11 moderate; Gruppo rivaroxavan: 37 mild, 3 moderate	Mild: Compressive hemostasis with gauze; moderate: unspecified
Galletti et al., 2020 [44]	Cohort study	Rivaroxaban	12	57	Yes (for 24 h)	No	Suture + compressive hemostasis for 30 min (+compressive hemostatis with gauze soaked with tranexamic acid for other 30 min if needed)	3	Compressive hemostasis with gauze soaked in tranexamic acid (+electrocauterization and additional sutures, if necessary)
Manor et al., 2021 [29]	Case–control study	Clopidogrel/ASA/DOAC/Warfarin/Combinations	72	Unspecified	No	No	Suture + gelatin sponge + Compressive hemostasis with gauze soaked in tranexamic acid for 20–30 min	4 (1 warfarin + DOAC; 2 Clopidogrel + ASA; 1 warfarin + clopidogrel); +7 controlli	Suture/suture + compressive hemostasis with gauze soaked in tranexamic acid
Buchbender et al., 2021 [21]	Case–control study	Antiaggregants/Dicumarols/DOACs	95	Unspecified	no	Unspecified	suture + compressive hemostasis with gauze soaked in tranexamic acid	1	Compressive hemostasis with gauze soaked in tranexamic acid

1. Search strings for CCDs

((dental AND implant AND haemophilia) OR (dental AND implant AND von AND willebrand) OR (dental AND implant AND thrombocytopenia) OR (dental AND implant AND bleeding AND disorder) OR (dental AND implant AND bleeding AND disorders) OR (oral AND implant AND haemophilia) OR (oral AND implant AND von AND willebrand) OR (oral AND implant AND thrombocytopenia) OR (oral AND implant AND bleeding AND disorder) OR (oral AND implant AND bleeding AND disorders) OR (implantology AND haemophilia) OR (implantology AND von AND willebrand) OR (implantology AND thrombocytopenia) OR (implantology AND bleeding AND disorder) OR (implantology AND bleeding AND disorders) OR (implant AND haemophilia) OR (implant AND von AND willebrand) OR (implant AND thrombocytopenia) OR (implant AND bleeding AND disorder) OR (implant AND bleeding AND disorders))

**2.**
1. Search strings for APBDs

((dental AND implant AND aspirin) OR (dental AND implant AND clopidogrel) OR (dental AND implant AND prasugrel) OR (dental AND implant AND ticagrelor) OR (dental AND implant AND antiplatelet) OR (dental AND implant AND antiplatelets) OR (dental AND implant AND antiaggregant) OR (dental AND implant AND antiaggregants) OR (oral AND implant AND aspirin) OR (oral AND implant AND clopidogrel) OR (oral AND implant AND prasugrel) OR (oral AND implant AND ticagrelor) OR (oral AND implant AND antiplatelet) (oral AND implant AND antiplatelets) OR (oral AND implant AND antiaggregant) OR (oral AND implant AND antiaggregants) OR (implantology AND aspirin) OR (implantology AND clopidogrel) OR (implantology AND prasugrel) OR (implantology AND ticagrelor) OR (implantology AND antiplatelet) (implantology AND antiplatelets) OR (implantology AND antiaggregant) OR (implantology AND antiaggregants) OR (implant AND aspirin) OR (implant AND clopidogrel) AND (implant AND prasugrel) AND (implant AND ticagrelor) OR (implant AND antiplatelet) OR (implant AND antiplatelets) OR (implant AND antiaggregant) OR (implant AND antiaggregants))

**3.**
1. Search strings for ACBDs

((dental AND implant AND warfarin) OR (dental AND implant AND sintrom) OR (dental AND implant AND heparin) OR (dental AND implant AND dabigatran) OR (dental AND implant AND rivaroxaban) OR (dental AND implant AND apixaban) OR (dental AND implant AND edoxaban) OR (dental AND implant AND anticoagulant) OR (dental AND implant AND anticoagulants) OR (dental AND implant AND noac) OR (dental AND implant AND noacs) OR (dental AND implant AND novel AND anticogulant) OR (dental AND implant AND novel AND anticogulants) OR (dental AND implant AND direct AND anticogulant) OR (dental AND implant AND direct AND anticogulants) OR (oral AND implant AND warfarin) OR (oral AND implant AND sintrom) OR (oral AND implant AND heparin) OR (oral AND implant AND dabigatran) OR (oral AND implant AND rivaroxaban) OR (oral AND implant AND apixaban) OR (oral AND implant AND edoxaban) OR (oral AND implant AND anticoagulant) OR (oral AND implant AND anticoagulants) OR (oral AND implant AND noac) OR (oral AND implant AND noacs) OR (oral AND implant AND novel AND anticogulant) OR (oral AND implant AND novel AND anticogulants) OR (oral AND implant AND direct AND anticogulant) OR (oral AND implant AND direct AND anticogulants) OR (implantology AND warfarin) OR (implantology AND sintrom) OR (implantology AND heparin) OR (implantology AND dabigatran) OR (implantology AND rivaroxaban) OR (implantology AND apixaban) OR (implantology AND edoxaban) OR (implantology AND anticoagulant) OR (implantology AND anticoagulants) OR (implantology AND noac) OR (implantology AND noacs) OR (implantology AND novel AND anticogulant) OR (implantology AND novel AND anticogulants) OR (implantology AND direct AND anticogulant) OR (implantology AND direct AND anticogulants) OR (implant AND warfarin) OR (implant AND sintrom) OR (implant AND heparin) OR (implant AND dabigatran) OR (implant AND rivaroxaban) OR (implant AND apixaban) OR (implant AND edoxaban) OR (implant AND anticoagulant) OR (implant AND anticoagulants) OR (implant AND noac) OR (implant AND noacs) OR (implant AND novel AND anticogulant) OR (implant AND novel AND anticogulants) OR (implant AND direct AND anticogulant) OR (implant AND direct AND anticogulants))
